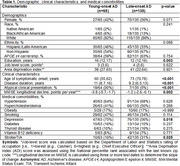# Socioeconomic deprivation and medical comorbidity burden associations with clinicopathologic characteristics in an ethnoracially diverse Alzheimer's disease autopsy series

**DOI:** 10.1002/alz70855_107360

**Published:** 2025-12-24

**Authors:** Christian Lachner, Sheina Emrani, Lindsey A. Kuchenbecker, Sara R Dunlop, Bailey D. Rawlinson, Erica Engelberg‐Cook, Josalen Ventura, Idaly Velez‐Uribe, Zhongwei Peng, Lauren Haydu, Dennis W. Dickson, Neill R. Graff‐Radford, Gregory S Day, John A. Lucas, Melissa E. Murray

**Affiliations:** ^1^ Mayo Clinic in Florida, Jacksonville, FL, USA; ^2^ University of Pennsylvania, Philadelphia, PA, USA; ^3^ Mayo Clinic, Jacksonville, FL, USA; ^4^ Department of Neuroscience, Mayo Clinic Florida, Jacksonville, FL, USA; ^5^ Wien Center for Alzheimer's Disease and Memory Disorders, Miami Beach, FL, USA; ^6^ Department of Laboratory and Medicine, Mayo Clinic, Jacksonville, FL, USA

## Abstract

**Background:**

Population aging is expected to increase the burden of Alzheimer's disease (AD) and related dementias (ADRD), yet up to 45% of ADRD risk may be modified. Disparities in ADRD burden among Hispanic/Latino and Black/African Americans suggests that socioeconomic deprivation and medical comorbidities may influence AD risk. The relationship between these factors, symptomatic age of onset, and neuropathologic findings in AD remains unclear. This study aims to investigate these associations in an ethnoracially diverse, neuropathologically diagnosed AD case series. Understanding these risk factors may help develop more effective AD risk reduction strategies.

**Method:**

We queried the Florida Autopsied Multi‐Ethnic (FLAME) cohort database for neuropathologically‐diagnosed AD cases with available age onset of cognitive symptoms, who self‐identified as Hispanic/Latino (*n* = 75) or Black/African American (*n* = 22). Cases were matched to non‐Hispanic White decedents (*n* = 101) by sex and year of birth. Medical records at the time of brain donation were retrospectively reviewed, assessing completeness for demographics, clinical characteristics, and medical comorbidities (Table 1).

**Result:**

The table shows a comparative analysis stratified by young‐onset AD (YOAD, onset <65 years) and late‐onset AD (LOAD, onset ≥65 years). YOAD cases had a significantly higher level of education compared to LOAD (*p* = 0.003). No differences were observed in sex*, APOE ε4* carriership, or Area Deprivation Index between YOAD and LOAD. However, YOAD cases had a longer disease duration and were more likely to present with atypical clinical features compared to LOAD (both *p* <0.001). YOAD cases also showed a faster rate of decline on MMSE compared to LOAD (*p* = 0.002), higher frequency of depression (*p* = 0.018), and lower frequency of stroke/TIA (*p* = 0.025). YOAD cases also had lower brain weight (*p* = 0.041), less cerebrovascular disease burden (*p* = 0.003) and higher neurofibrillary tangle density in association cortices (*p* <0.001) compared to LOAD cases.

**Conclusion:**

Socioeconomic deprivation and most medical comorbidities were not associated with AD symptomatic onset. Despite having higher education levels and lower cerebrovascular burden, YOAD decedents experienced faster cognitive decline but a longer disease course, with more frequent atypical clinical presentations and depression compared to LOAD. Further discussion will address the topographic distribution of tangle and plaque pathology.